# HUG: A Compassionate Approach to Designing for Wellbeing in Dementia Care

**DOI:** 10.3390/ijerph20054410

**Published:** 2023-03-01

**Authors:** Cathy Treadaway, Abdul Seckam, Jac Fennell, Aidan Taylor

**Affiliations:** 1 CARIAD, Cardiff Metropolitan University, Cardiff CF5 2YB, UK; 2 Department of Architecture and the Built Environment, University of the West of England, Bristol BS16 1QY, UK

**Keywords:** dementia, design, hug, person-centered, prudent care, psychosocial, anxiety, social prescribing, compassion

## Abstract

Design can improve the quality of life of people living with dementia but creating successful design solutions is not simple, due to the complexity of the medical condition, and the ethical considerations of including those affected in design research and evaluation. This article describes research involving an interactive product, ‘HUG’, developed from academic research, to support the wellbeing of people living with advanced dementia, which is now commercially available. People affected by dementia were included at every stage in the design research process. The evaluation of HUG took place in both hospital and care home contexts with 40 participants living with dementia. In this paper, a qualitative hospital study is described, in which patients received a HUG on prescription. Findings reveal that although HUG was rejected by some, those patients who did accept it benefitted significantly. Not only did the device reduce distress, anxiety and agitation but it also helped with patient compliance in medical procedures, aspects of daily care and enhanced communication and socialisation. The Alzheimer’s Society’s accelerator partnership funding has enabled this product to be manufactured and made commercially available so that the benefits of this academic design research can be made more widely available to people living with dementia.

## 1. Introduction

Dementia has been described as a ‘wicked design problem’ [1] (p. vii). The medical condition is complex, the numbers of those affected huge and each person’s lived experience unique. Individuals, families and society as a whole are impacted by the condition with numbers diagnosed globally in excess of 55 million people [2,3]. Dementia is associated with over 100 different neurological diseases of the brain, impacting a range of brain functions including memory, cognition, behaviour, orientation and perception. It is a progressive degenerative condition for which there is currently no cure [4]. Following diagnosis, a person may live with dementia for many years and so it is vital to find ways to help people to live well and maintain their quality of life [5].

Designers have an important role to play in designing products, services and spaces that can support the wellbeing of people living with dementia [1,6]. Despite the ‘wickedness’ of the problem, there is increasing interest in the design community to generate new designs for dementia care [7]. There is also support from governments and charities to stimulate and advise on designing practical solutions to help people live well [8,9]. Creating designs that are intuitive to use, appealing and practical requires a deep understanding of the issues faced by those affected by dementia. There is a growing consensus that the best approach is to include people with lived experience of the condition in the design research process [1,6,7,10]. Their insights into the challenges faced by those living with dementia can inform appropriate and novel designs that can help improve quality of life [11]. 

Academic research is contributing important knowledge to this field; however, the resulting design outputs frequently fail to be commercialised and so never actually impact the lives of the intended users as they are unable to purchase them. Considerable investment funding is required to deliver a successful proven outcome that is compliant with the stringent regulatory demands of the medical and care sector [12].

**Figure 1:**
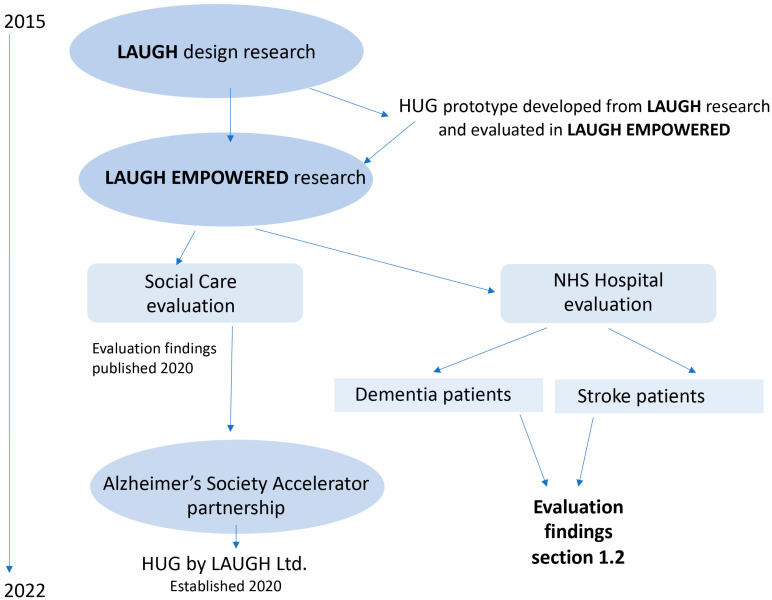
Timeline and structure of the research and product.

This paper reports on UK academic research that has resulted in the creation of a product that integrates embedded technology and is designed to reduce anxiety and provide comfort. It draws from two government-funded academic research projects, LAUGH and LAUGH EMPOWERED, and an accelerator partnership award from the Alzheimer’s Society which enabled the product to be commercialised. The timeline of the project is illustrated in Figure 1. The second half of this paper focuses on a qualitative evaluation of the product in a hospital context. It describes the impact on patient wellbeing as reported by medical staff (Section 1.2).

**Figure 2:**
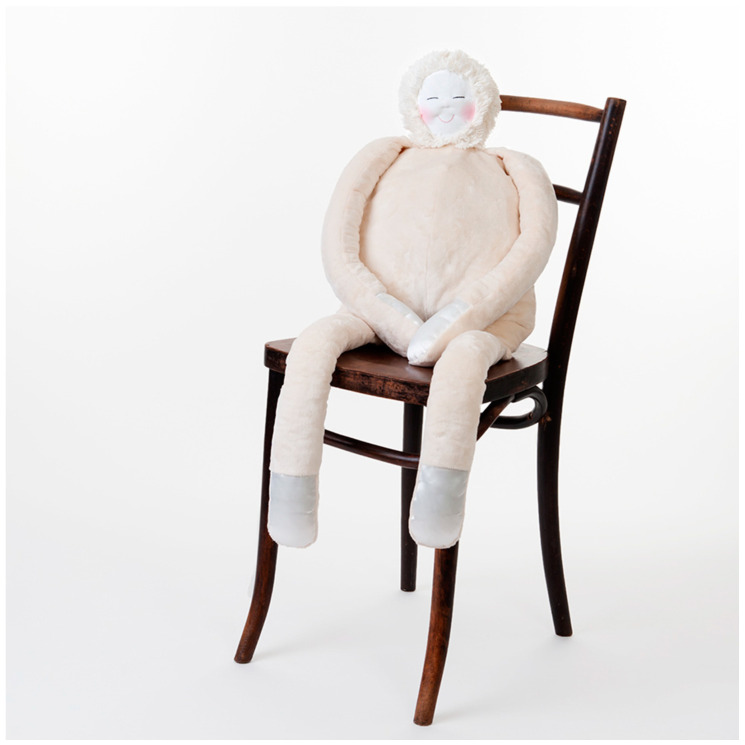
HUG by LAUGH^®^ therapeutic comforter.

HUG is a soft therapeutic calming device used to support the wellbeing of people living with dementia. It is designed to be cuddled and has a plush textile outer body with weighted arms and legs (Figure 2). Its soft inner cushion contains a programmable electronics module, which provides the pulsing sensation of a beating heart and plays a person’s personalised playlist of music [13]. Soft, comforting products such as Paro^®^, sensory dolls, teddy bears and plush pets have been found to provide comfort for people living with dementia [14,15]. The Ludic Artifacts Using Gesture and Haptics (LAUGH) design research that led to the development of HUG involved participants living with dementia at every step in the design process, from concept development to the packaging design of the final manufactured product [16]. With the support of the Alzheimer’s Society, UK and Welsh government funding, the university research team has been able to scale up a successful design concept into an award-winning [17] product. Customer and user feedback continues to evidence the benefits of HUG to support wellbeing and quality of life, not just for people living with dementia but with a range of medical and psychosocial needs [13,18]. 

### 1.1. LAUGH Academic Research

**Figure 3:**
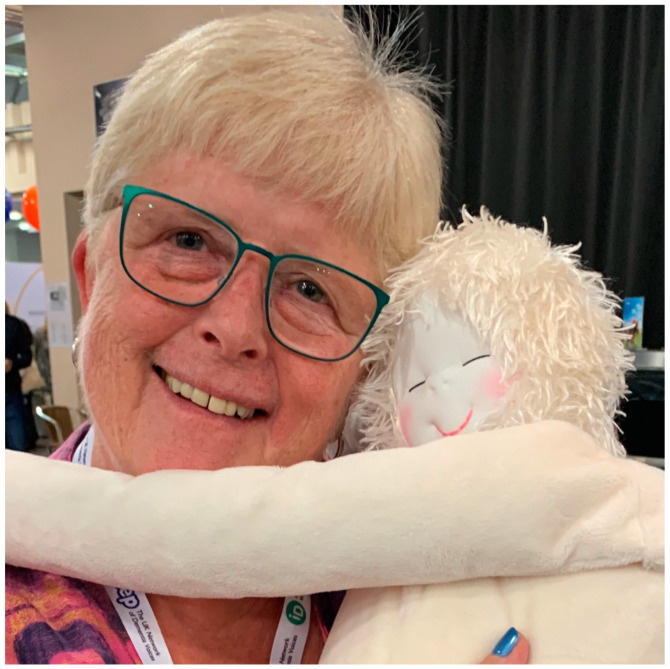
Person living with dementia holding a prototype HUG. Photo C. Treadaway.

HUG was one of six prototype playful objects designed during the Arts and Humanities Research Council (AHRC)-funded LAUGH research (2015–2018) [16,19]. This project sought to find ways to comfort, stimulate and engage people living with advanced dementia. This was achieved through the design and development of age-appropriate playful objects that could be used in their care to improve quality of life. The LAUGH research involved people with lived experience of the disease, their families and carers, health professionals, technologists and academic researchers (Figure 3). Designs were developed and evaluated with people living with dementia in two residential care homes in Wales. HUG was originally designed for a specific person living with advanced dementia in one of these care homes. The LAUGH research evaluation evidenced significant improvements to her health and wellbeing following the use of HUG over a period of 3 months [20]. On the strength of the findings from the study, the Welsh government funded LAUGH EMPOWERED (LE): a much larger evaluation study involving 40 participants in social care and NHS hospital contexts. The LE care home evaluation corroborated findings from the initial study with 87% of those who used the product over 6 months showing an improvement in their wellbeing and over half of those also showing an improvement in their cognitive and functional ability [21].

The LE hospital study took place just before and during the global pandemic (2019–2021) and due to staff pressures and hospital restrictions on research, it was not possible to replicate the protocol used in the care home study as had originally been intended. Rather than abandon the study altogether, HUGs remained with patients during the pandemic, and a qualitative study was undertaken once restrictions eased. The following sections report on this evaluation process and set out the findings from this study. 

### 1.2. LAUGH EMPOWERED NHS Hospital Study

Agitation, anxiety and distress are common symptoms presented by patients living with dementia. Finding ways to alleviate a person’s distress and reduce anxiety, without resorting to medication which may interfere with other aspects of the condition being treated, are desperately needed, particularly in hospital contexts [22]. Patients who are admitted to hospital with dementia are often in crisis. This may be as a result of comorbidities that require them to be hospitalised or because progression of their dementia has reached a critical stage and they need specialist medical care. These patients may have a diagnosis of one specific form of dementia (e.g., Alzheimer’s disease), post-stroke cognitive impairment (PSCI) or a combination of neurodegenerative diseases of the brain. Patients who participated in the LE NHS study were critically ill and were either (i) cognitively impaired as a result of a stroke, (ii) exhibiting extreme dementia symptoms requiring specialist care or (iii) in the most advanced stage of the disease. 

The study began in October 2019 as an NHS Service Improvement Evaluation study when 20 HUGs were delivered to the Stroke Rehabilitation Centre (SRC) at Llandough Hospital. Although this was prior to the pandemic, the SRC was undergoing extreme winter pressure and staffing issues. As a result, only nine HUGs were evaluated during the pre-pandemic period November 2019–March 2020. In early March, as the first wave of the pandemic began, the remaining HUGs were transferred for use with patients in the dementia wards. At this point, no visitors were permitted in the hospital and the hospital was in strict lockdown measures, prioritising patients with COVID-19; a halt was called to all research by the health board. Those patients using HUGs at this time continued to do so, but the university research team was unable to provide any technical support or undertake any evaluation research. It was inevitable that undertaking the intervention during a global pandemic would influence findings in this study. Staff time was stretched, staff morale low, anxiety levels high and there were long delays in communication. It was not possible to undertake staff evaluation interviews until a year after the initial intervention, due to the NHS moratorium on granting research approval. Despite these difficulties, qualitative interviews with a range of medical staff from the multidisciplinary teams (MDT) in the SRC and dementia wards were eventually undertaken in April 2021.

## 2. The Research Protocol—(Materials and Methods)

**Figure 4:**
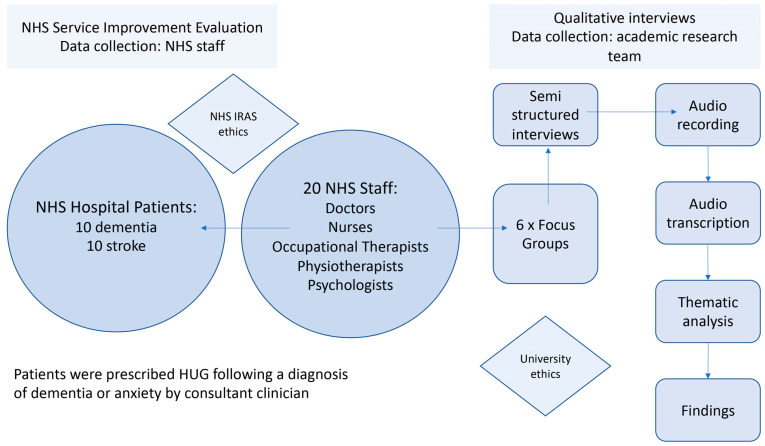
Research methods used in qualitative study in a hospital context. HUGs were prescribed to 20 NHS patients by consultant clinician and observations were made by MDT caring for them.

The overarching aim of the research was to improve the lives of people living with dementia: particularly those in the advanced stages of dementia and those living with post-stroke cognitive impairment (PSCI). Prior research found clear evidence of the benefits of HUG with people living with dementia in residential care [20,21]. Findings from the study reported here demonstrate how this might be affected by the hospital context, patient diagnosis (stroke/dementia) and the critical nature of the conditions being experienced by patients admitted to the hospital (Figure 4). Post-intervention qualitative data were gathered from NHS health professionals (n = 20) via a series of interviews which were undertaken as small groups (max 3) or individually, in order to accommodate the busy schedules of the multidisciplinary team (MDT) NHS staff members who participated. Ethical consent for the qualitative interviews was gained via the NHS IRAS process in May 2020 (NHS IRAS ethics project ID reference:273653). 

A qualitative interpretivist grounded practical theory methodology [23] underpinned by a compassionate design approach was used in this research [24]. This methodology was developed and tested in prior LAUGH research and has been widely published and used by other design researchers in the field [25]. The approach is phenomenological and focuses on capturing data relevant to the lived experience of participants, their personal reflection on that experience and their empathic perceptions of the experience of others within their care (informed by their intimate personal knowledge of that person and their observed embodied responses) [26]. The purpose of using this methodological approach has been to uncover the unique and individual responses of users to HUG. The interviews provided individual reflections from NHS staff on the impact of using HUG with patients in their care in a hospital context. 

NHS IRAS regulations specified the conditions set out for undertaking the research interviews, their required anonymity, data protection, means of collecting data and number of participants to be recruited. No interviews were undertaken ‘in person’ due to COVID-19 regulations and all took place via Microsoft Teams. Interviews were audio recorded for accuracy and later transcribed by the research team and anonymised. All specialisms in the multidisciplinary teams were represented and these included doctors, nurses, speech and language therapists, psychologists, physiotherapists, occupational therapists and technicians. Participants (n = 20) were recruited, and their ethical consent collected by the NHS research nurse in the Stroke Rehabilitation Centre, under the direction of the NHS Chief Investigator. Interviews were undertaken by two members of the academic research team. All participants recruited to the study had experience in caring for a patient who had received HUG as part of their in-patient care at the hospital.

**Figure 5:**
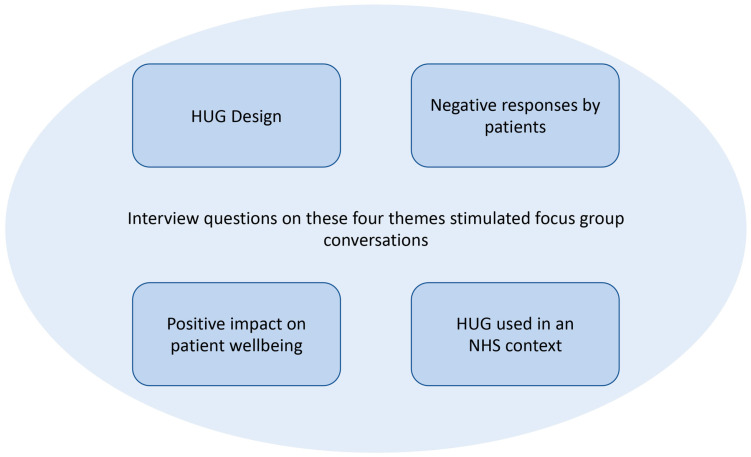
Themes identified to shape focus group questions and guide conversations.

A data analysis was undertaken by two members of the research team. Key themes crucial to the study were identified prior to the analysis phase (Figure 5): (1) HUG design, (2) evidence of improvement in patient wellbeing, (3) difficulties and negative responses to using HUG (patient and staff) and (4) themes arising from using HUG in an NHS hospital context. 

### Limitations and Constraints

Data collected in the qualitative interviews reflect the participants’ personal experience of how HUG impacted the patients they cared for in a hospital context. It must be noted, however, that three important factors impacted the validity and reliability of the data:

(1) the gap in time between the intervention and qualitative interviews;
(2) a negative bias of NHS participants to the research due to the stress of research reporting (financial evidence) for the funders;
(3) lack of time available in NHS staff work schedules to collect data.

In any qualitative study, factors surrounding the research process, context and chronology can inevitably introduce bias. This study was undertaken during unprecedented times, in the midst of a global pandemic that has significantly disrupted, challenged and exhausted NHS staff. Government and NHS regulations due to COVID-19 disrupted the research plan, and there was a huge and unforeseen gap of 12 months between the intervention and qualitative staff interviews. Some participants struggled to remember details as a result.

From the initial phase of the intervention in late October 2019, the SRC was facing significant staff shortages and the usual winter pressures on the NHS. When interviewed, participants focused heavily on their lack of time to engage wholeheartedly with the intervention. In order to make it possible to compare data from this NHS study of HUG with the care home context study, staff were required to use the same metrics. Staff were trained to collect data via the Pool Activity Level tool (PAL) [27,28] and were asked to use an adjusted Bradford Dementia Wellbeing Profile [29] to collect baseline and post-intervention patient data. These measures were not used consistently during the intervention due to lack of staff time (and their confidence in using them). Since these data are incomplete and unreliable, they have been excluded from these findings. The added pressure for staff attempting to undertake PAL and Wellbeing data collection when they were already overstretched resulted in some staff admitting to having negative preconceptions about HUG and its impact on patients.

Despite its limitations, the NHS study was useful to inform the development of HUG as a commercially viable product. The design was modified to adhere to NHS regulations for infection control and safety and tested in a ‘real world’ clinical setting during extreme conditions resulting from the pandemic. Adjustments were made to the design of the HUG body so that it could be more easily laundered and to the electronics unit to ensure there was no conflict with other medical devices. 

As well as providing useful design data, the qualitative interviews also provided evidence of the positive impact of HUG on patient care, as well as highlighting the potential barriers to its implementation as a service improvement in the NHS.

## 3. Case Stories

An analysis of the qualitative interviews with those NHS staff who had used HUG with patients revealed many positive case stories. Although not every patient who was prescribed HUG accepted it, those that did were found to benefit significantly. The following case stories describe the positive impact HUG had on specific patient wellbeing and care provision. They have been selected from the narrative data to provide examples of how HUG impacted (1) someone living with dementia; (2) a patient with PSCI; and (3) a cognitively impaired patient who was suffering from loneliness, anxiety and agitation.

1. A patient living with advanced dementia.

An occupational therapist described a patient that she had been working with in the dementia ward. The patient had been admitted during the first wave of the pandemic and had experienced social isolation for many months. HUG provided comfort and emotional release to support her wellbeing.

*One lady that I’ve been working with quite closely absolutely adores hers. It’s been such (and still is) a positive experience for her. She’s quite…... she can’t communicate verbally at all. It’s very hard to get her to actually focus on you, but she’s looking for sensory input quite a lot, so when I introduced the HUG doll (her field of vision is quite bad) we’re not sure how much she can see, I think it was the weight—the response I had from her was fantastic!*

*I popped the arms round and the legs round so she could feel the weight and feel the pressure on her and she literally burst into tears and howled, it was so sweet. And this was during the first wave of the pandemic, so she’d lacked any sort of proper physical comfort and I think her sort of crying was like a release. You know, because ‘oh this is what I need’ or this is what I needed. So that was a fantastic response. We used the HUG doll a lot with her. She clings, she likes it to be very close to her during very personal care. That’s giving comfort, so it’s almost like she’s not without it very often. She really likes chocolate and likes to share it so it gets washed quite a lot which is fine. It’s absolutely fine, it’s washing fine. And we just air dry it overnight, so it’s been a pleasure to work with that lady and HUG doll. It’s been an amazing response from her.*

*They really have a positive impact on the patients that I’ve been working with.*

(Occupational Therapist)

2. A patient with sudden onset vascular dementia following stroke.

When patients are hospitalised, they are often frightened, agitated and disorientated due to the health crisis for which they have been admitted; they have left the safety of home and entered an alien environment, full of unfamiliar people and sensory experiences. For someone experiencing cognitive impairment, sensory loss, dementia or delirium this can be devastating. Finding ways to calm patients, so that medical procedures can take place, is vital for positive health outcomes and crucial for their care and wellbeing. A doctor described a patient experiencing sudden onset vascular dementia following stroke and explained how HUG provided her comfort in the acute phase of her condition, reducing her distress and enabling her to receive medical care.

*[There was one lady]…. who had been very, very independent, fiercely independent woman and had such a bad stroke that it effectively made her behave as if she had advanced dementia overnight. And she was really, really, tricky to manage because she had no insight and was very distressed, by what was going on. Every time we tried to do anything with her, she would scream and cry. It was really hard to get through to her. Literally, you put HUG on and almost immediately she was like a different woman. She found that very soothing, it was definitely more effective when the heartbeat was turned on but ……It became less effective over time, and she became less attached to it over time, so initially she was really invested in it and cwtched (‘cwtched’ is a Welsh word that means ‘cuddled’ or ‘hugged’) in with it. And then a week later, 2 weeks later, it would be next to her on the bed rather than on her. But that seemed to make a real positive difference to her, at least initially, I think to manage that distress right in the really acute phases, and I don’t think that was coincidence. I think that was the effect of the HUG……*

(Doctor)

3. A patient who was lonely anxious and distressed,

Anxiety distress, agitation and boredom can accompany hospitalisation for many patients with cognitive impairment or dementia. Wards are busy environments and there is little time for staff to stop and chat with patients when they are lonely, bored or lacking self-confidence.

HUG was described by a doctor as: *sort of something to cling to, a bit of comfort, so [they] gained a bit more confidence on the ward and were a bit more orientated.*

*[The patient] was a lady who liked company, I think. And if she could, she would have somebody sat next to her all of the time, passing her things, having a chat to her. She didn’t really like to be left alone, and when she was left alone, she would shout repeatedly. She would bang the table. All to gain attention, to get somebody’s attention. When people were actually doing things with her, she wasn’t as bad. So, what we were using the device for was almost like a reassurance, like a comfort.*

Interviewer: *As a companion?*

Doctor: *Yeah. A companion yeah! She was just much more settled if she had the HUG with her. That’s why I say, when she was in bed. Because it seemed to be that if she was alone, and there was nobody with her; and to be honest you just can’t, we don’t have the staffing level to be persistently with somebody like that, constantly.*

## 4. LAUGH EMPOWERED Analysis

All patients that were included in the intervention had undergone a physical or mental health crisis as a result of stroke or dementia. The medical team purposefully selected patients considered likely to benefit from the intervention. These were those who were assessed as having cognitive impairment or dementia and also presented with anxiety and agitation. Patients’ families were consulted, and ethical consent gained for their participation in the evaluation by the prescribing consultant clinician. HUG was provided on prescription to these patients, along with a specific associated care plan. 

**Figure 6:**
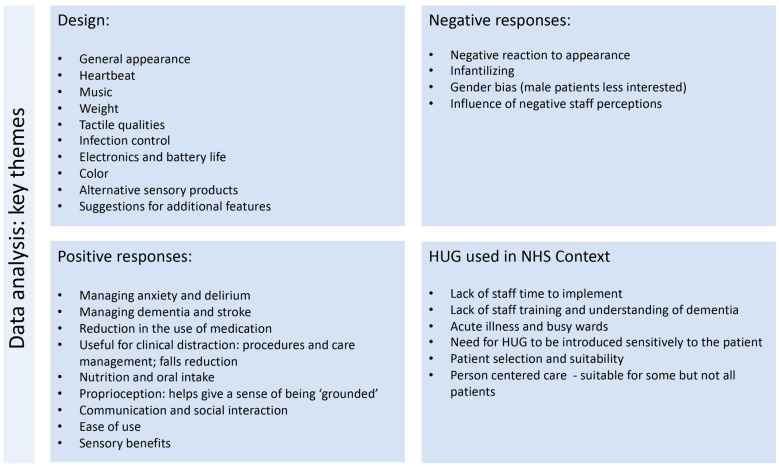
Key themes identified from analysis of the interview transcripts.

An analysis of the data collected from the NHS staff qualitative interviews provides a rich picture, detailing the complexities of introducing a therapeutic playful object into an NHS context with critically ill patients (Figure 6). The interview data provide the consensus that HUG was beneficial for some patients, but not all. Where patients responded well, using HUG led to a significant positive change in their wellbeing, lowering anxiety, providing comfort and making possible medical treatment that they required but had previously resisted. One doctor commented on a female patient in her care who had been prescribed HUG:

*It did have a really profound effect on her……. It really was a distressing case, the whole team struggled with her, it was because it was such a dramatic change, and we were so struggling to get through to her. And so it was nice being able to have something that we could do that did give her a bit of comfort and did make it seem less frightening for her.*

However, for some patients HUG was rejected immediately (sometimes vehemently) and found to have little use in their care or impact on their wellbeing. As one physiotherapist described:

*I remember, certain patients not taking to it at all, it actually sort of heightening some of their sort of behavioural sort of things in regard to aggressiveness towards it really…. um, so that was probably 50% and the other 50% just absolutely loved it and sort of really, really took to it, and wouldn’t do anything without it then.*

Although the data present a mixed response to HUG, all the doctors interviewed considered it to be a positive asset in the range of treatment options available for distressed cognitively impaired patients. As one doctor noted, as a non-pharmacological intervention, HUG has no detrimental side effects on the patient, can be introduced quickly and taken away if not found to be suitable:

*I’m really not that keen on using medication for people who are delirious or who are cognitively impaired or need reassuring, so I would definitely love to have one of those on my ward and be able to use it for people, because I have seen that it does work sometimes. I just feel, if you try and it fails you haven’t lost anything, like, it’s not a problem, is it? You just say, OK well, that didn’t work, so I will try something else. It’s not like stabbing them with haloperidol. I don’t know. I just think it’s a nice thing to have, as a nonpharmacological way of managing people. So, I would love to have one on my ward.*

## 5. Key Findings from the Study

Data collected from the qualitative interviews contained a far greater incidence of comments about positive experiences of patients with HUGs than it did descriptions or reflections about negative experiences. In summary, the positive benefits of using HUG in hospital care were evidenced by members of the multidisciplinary team in the following ways:

1. Providing comfort, calming and reassuring;
2. Enhancing communication and initiating conversations;
3. Providing company and purpose for life;
4. Improving oral intake: drinking, feeding and taking medication;
5. Alternative to medication (reducing risk of falls);
6. Enabling medical procedures and personal care to take place;
7. Clinical distraction—modifying behaviour (tapping, shouting, distress);
8. Proprioception and feeling ‘grounded’.

The implementation of HUG within individual care packages was impacted by the attitudes and preconceptions of those staff involved. Although it is not unusual for cognitively impaired patients to be admitted to hospital with soft toys and comforters, for some patients, being given a HUG was perceived to be infantilising. Staff members were also challenged by the concept of a person being given what they considered to be a ‘toy’. One of the doctors noted staff uncertainty about using HUG with patients:

Doctor: *I think some people thought it was a bit odd. ‘Why are we using this?’ Well, it might be a little bit unusual here but I’m sure in care homes it’s not as unusual for patients to have something to carry and cwtch (cuddle), ‘look after’ almost.*

Interviewer: *Did they feel it was infantilising?*

Doctor: *Yeah, I think there was a sense of that, there was a sense of well, this is just a bit odd. But then at the same time, we do get quite a lot of patients with cognitive impairment who come with their own soft toys and stuff, from home, you know.*

Some interviewees felt that personal negative perceptions of members of the staff about HUG impacted the way it was introduced to patients. One nurse commented:

*A lot of it was down to the nursing and healthcare staff when they are providing that care, and things. It’s only certain staff would initiate that (using HUG) and chat. So, some would say: ‘Well what’s this?’ and throw it to the side.*

The data revealed the importance of a person-centred approach to implementing HUG within a care plan. In this study, HUG was used with more women than men, and with patients more likely to present with agitation and anxiety, who tended to be older. Understanding the needs and preferences of individual patients was observed to be vital for successful outcomes. Equally important was the attitude of the staff involved in introducing HUG to a patient, and the way in which this was done. Staff training in cognitive impairment, dementia and delirium as well as adequate time spent with the patient were identified as two major factors essential for positive outcomes with using HUG in an NHS hospital context. 

The interview data revealed ways in which the successful implementation of HUG in a clinical context required the following of staff:

- understand cognitive impairment, dementia and delirium and how this impacts patient perception of the world;
- appreciate that soothing and playful objects can assist in care;
- understand the personal preferences of the patient;
- suspend personal views and have a willingness to try something new;
- understand ways of introducing care objects with compassion.

## 6. Discussion

Findings from the LAUGH EMPOWERED (LE) hospital study complimented those from the earlier LE care home study. Although it is not possible to make direct data comparisons, the qualitative LE hospital study illuminated the ways in which it is vital to understand the context in which the product is used and the management and training of those implementing care. The LE care home study used evaluation tools [27,28,29] that were able to provide clear insights into the impact on an individual user’s wellbeing and improvement in cognitive and functional abilities. The timing of the hospital study (which was undertaken during the pandemic) made the use of these tools to collect data impossible due the additional time pressures on NHS staff caring for patients with COVID-19. The qualitative interviews, however, were able to provide nuanced and detailed descriptions of patient and staff responses in ‘a real-life context’. This was invaluable for the development of the product design and provided useful guidance into how the product should be introduced to and used with patients. Useful ideas about improvement to the design of the product and innovative suggestions about how HUG might be incorporated into care activities were also offered by staff in the interviews. 

**Figure 7:**
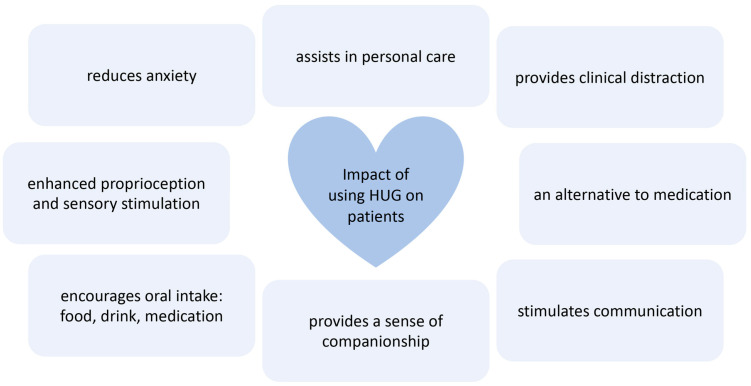
Findings: reported benefits of using HUG in an NHS hospital context.

The study revealed how important it is to understand a patient’s individual preferences and psychological needs. HUG needs to be introduced sensitively to a patient with cognitive impairment. To do this requires staff time and commitment, and in a busy hospital environment this can be a challenge. However, for those patients who responded well to HUG, their hospital experience was transformed, anxiety was reduced, medical procedures were less stressful and they required less staff time as they were happier, less agitated and calmer. In addition, less medication was required in their care, resulting in the potential for fewer falls with the many associated healthcare cost savings that this reduction implies (Figure 7). 

**Figure 8:**
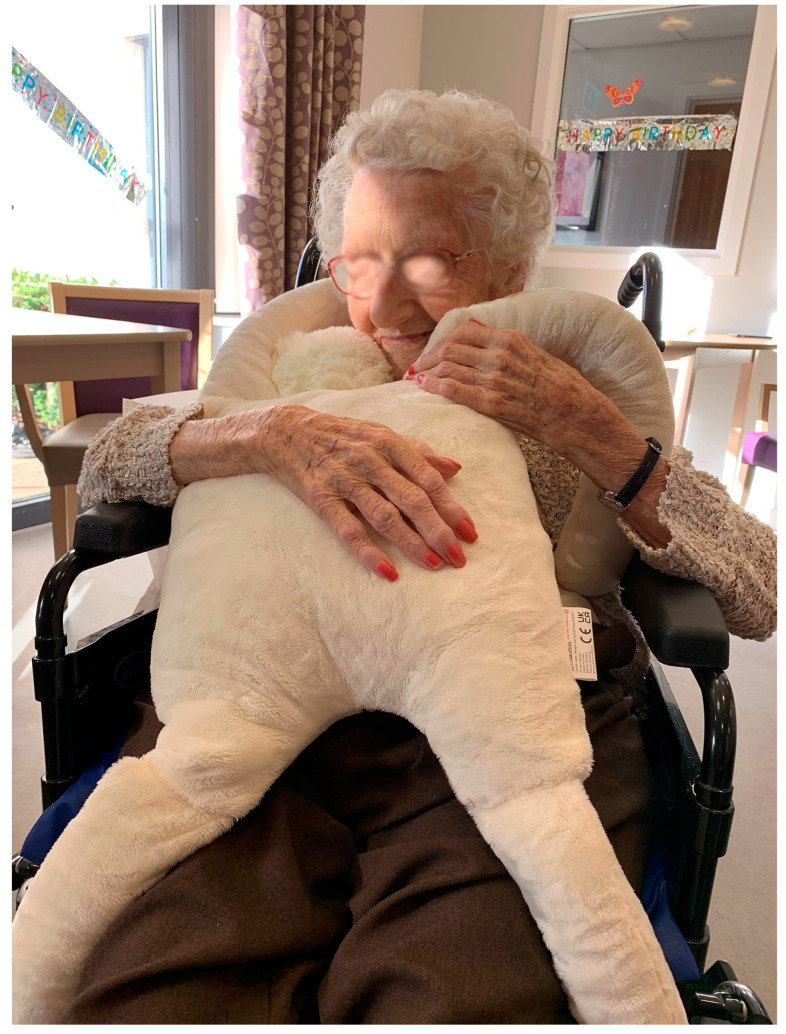
Person living with dementia using a manufactured HUG.

The success of HUG in improving the quality of life of people, witnessed during the LAUGH and LAUGH EMPOWERED studies, and media interest in the UK [30,31] gave the research team impetus to commercialise the design [13]. Following the LE NHS research, HUGs have been purchased by health boards and hospitals across the UK for use with patients living with a variety of medical and psychological conditions (Figure 8). Further research is currently underway in several of these NHS contexts where HUG is now being received by patients ‘on prescription’.

## 7. Conclusions

There is a growing need for appropriately designed products and services to help people ‘live well’ with dementia, particularly as it is a degenerative condition that can affect people over many years [6,11]. Designing ways to preserve a person’s sense of self, reduce anxiety and agitation and bring comfort, joy and pleasure are vital if a person’s quality of life is to be maintained. Technology enables products and services to be developed that can be easily personalised so that they can be manufactured at scale and yet meet the individual needs of the user [32,33].

Everyday interactive products, such as HUG, have the potential to support the health and wellbeing of people living with dementia throughout the course of the disease. Ensuring that the technology is unobtrusive and intuitive to use is vital if it is to add value to the user experience of the product. Even more important is that the design features it provides are relevant and beneficial to the person living with dementia and do not simply add layers of complexity resulting in cognitive overload for the user. Understanding what a person ‘needs’ when they experience communication and memory difficulties can be complicated, as is knowing what ‘living well’ means when a person is unable to express clearly how they are feeling. Design and evaluation processes can be tricky when dealing with these kinds of ‘wicked design’ problems [1].

The design research that led to the development of HUG was underpinned and guided by a compassionate design methodology [24,33]. This approach places loving kindness for the individual at the heart of the design process. It challenges the designer to prioritise the sensory, personalised and connecting aspects of a design concept, in order to help keep the person living with dementia living ‘in the moment’ (not relying on memory), to maintain their dignity (enhance their sense of self) and remain connected socially and to the world around them. The approach is ‘asset’ based, working with what a person can do and building on their personal strengths and abilities. For someone in the advanced stages of the disease, this may involve very simple sensory stimulation through the choice of appropriate materials, incorporating their favourite music or stimulating an opportunity for conversation to create connection. 

The successful development of HUG from academic research would not have been possible without the help and involvement of people living with dementia, care professionals and a multidisciplinary team of researchers. The involvement of the Alzheimer’s Society in the research and product development has been invaluable and has enabled families affected with dementia to contribute their feedback and ideas to improve the user experience. The research has also contributed to their recently published design guidelines, produced to encourage the industry to develop new products and services to improve the quality of life of people living with dementia [11].

This study contributes qualitative evidence to illuminate the wellbeing benefits of using therapeutic objects with patients living with dementia and cognitive impairment in a hospital context. Ongoing NHS research continues to evaluate the use of HUG with patients with other medical and psychological conditions in the hospital, residential care and community environments in the UK.

## Data Availability

Restrictions apply to the availability of these data. Data were obtained in partnership with the NHS and are available from C. Treadaway via Cardiff Metropolitan University only with the permission of the NHS. Further information is available: https://figshare.cardiffmet.ac.uk/search?q=LAUGH (accessed on 26 February 2023).
